# CaMKIIα neurons in the anterior insular cortex regulate attention behavior in mice

**DOI:** 10.3389/fncir.2023.1197541

**Published:** 2023-07-04

**Authors:** Yingping Ma, Shaofei Jiang, Xin Zhao, Shen Li, Liping Chen, Zhe Zhao, Wei Shen, Yan Wu, Haitao Wu

**Affiliations:** ^1^School of Basic Medical Sciences, Anhui Medical University, Hefei, Anhui, China; ^2^Department of Neurobiology, Beijing Institute of Basic Medical Sciences, Beijing, China; ^3^Key Laboratory of Neuroregeneration, Co-innovation Center of Neuroregeneration, Nantong University, Nantong, China; ^4^Chinese Institute for Brain Research, Beijing, China

**Keywords:** insular cortex, CaMKIIα, 5-choice serial reaction time task, attention, impulsiveness

## Abstract

**Introduction:**

The insular cortex is involved in multiple physiological processes including working memory, pain, emotion, and interoceptive functions. Previous studies have indicated that the anterior insular cortex (aIC) also mediates interoceptive attention in humans. However, the exact cellular and physiological function of the aIC in the regulation of this process is still elusive.

**Methods:**

In this study, using the 5-choice serial reaction time task (5-CSRTT) testing paradigm, we assessed the role of the aIC in visuospatial attention and impulsiveness in mice.

**Results:**

The results showed a dramatic activation of c-Fos in the aIC CaMKIIα neurons after the 5-CSRTT procedure. *In vivo* fiber photometry revealed enhanced calcium signaling in aIC CaMKIIα neurons when the mice responded correctly. In addition, chemogenetic suppression of aIC CaMKIIα neurons led to increased incorrect responses within the appropriate time. Importantly, pharmacological activation of aIC CaMKIIα neurons enhanced their performance in the 5-CSRTT test.

**Discussion:**

These results provide compelling evidence that aIC CaMKIIα neurons are essential for the modulation of attentional processing in mice.

## Introduction

Attentiveness is usually described as selecting the right information, reducing distracting information, and enhancing performance (Ghosh and Maunsell, 2021). There are several psychiatric disorders that are characterized by cognitive deficits, such as schizophrenia (Sui et al., 2018), attention deficit hyperactivity disorder (Fleming et al., 2017), and bipolar disorder (Reinen et al., 2018). Therefore, it is particularly important to study the mechanisms underlying attention deficiency-related diseases. Increased understanding of attentional networks has been a growing interest in the past decades (Petersen and Posner, 2012).

Neuroimaging, electroencephalogram and pharmacological studies have revealed that the insular cortex (IC) is involved in specific aspects of attention, such as decision-making (Ghareh et al., 2022), anxiety (Stein et al., 2007), and mood (Roy et al., 2017). The IC is one of the most complex anatomical hubs in the mammalian brain, including the anterior insular cortex (aIC), medial insular cortex (mIC) and posterior insular cortex (pIC) (Gehrlach et al., 2020). The pIC is mainly connected to the sensorimotor integration area, which plays a critical role in pain regulation (Gehrlach et al., 2019). The aIC is linked to the limbic region and associated with cognitive functions, such as behavioral motivation (Deng et al., 2021). Recent studies in humans have shown that the aIC mediates interoceptive attention which is associated with increased activation of the aIC (Wang et al., 2019). However, the function of the aIC in visual attention is not fully understood.

There are several behavioral tasks used to measure the attention of animals, such as the 5-choice serial reaction time task (5-CSRTT), 5-choice continuous performance task (5C-CPT) (Lustig et al., 2013) and sustained attention task (SAT) (Varalta et al., 2014). Among them, the 5-CSRTT behavior paradigm is the most well-known and extensively employed model (Bari et al., 2008). Adapted from the continuous performance attention task used in human studies, the 5-CSRTT can be used extensively to analyze attention and impulsivity in rodents (Counotte et al., 2011; Kim et al., 2016; Tan et al., 2018; White et al., 2018).

In this study, we used the 5-CSRTT behavior paradigm to investigate the potential role of the aIC in regulating attention and impulsivity in mice. We first identified the cell type of activated neurons in the aIC after completing the 5-CSRTT by immunofluorescence colocalization analysis. Next, we detected the activity of CaMKIIα neurons in the aIC during the 5-CSRTT by fiber photometry recording *in vivo*. Furthermore, we examined the effects of chemogenetic inhibition of aIC CaMKIIα neurons on the performance the of 5-CSRTT. We also investigated the effects of activation of aIC CaMKIIα neurons on attention performance in mice.

## Materials and methods

### Mice

Male C57BL/6 mice (7–8 weeks old) were purchased from SPF (Beijing) Biotechnology Co., Ltd. (Beijing, China). Given the differences between 5-CSRTT behavior in males and females (Bayless et al., 2012), only single-sex groups were tested in our study. The mice were group-housed (four to six per cage) on a 12-h light/12-h dark cycle in a temperature- and humidity-controlled housing facility. All animal experiments were conducted in accordance with protocols approved by the Institutional Animal Care and Use Committee of the Beijing Institute of Basic Medical Sciences (SYXK 2019-0004).

### AAV viruses and drug

To observe the response pattern of CaMKIIα neurons in attention behavior, we injected adeno-associated virus (AAV) expressing an excitatory DREADD (artificially designed receptors that are only activated by artificially designed drugs, hM3Dq) or inhibitory DREADD (hM4Di) into the insula to modulate the activity of the IC. The drug used in this study was clozapine N-oxide (CNO) (BrainVTA, Wuhan, China), which was dissolved in 0.9% physiological saline. CNO (0.3 mg/kg) was intraperitoneally injected into mice. AAV2/9-CaMKIIα-GCaMP6s (5.31E + 12 vg/mL, PT-0110), AAV2/9-CaMKIIα-EGFP (5.08E + 12 vg/mL, PT-0290), AAV2/9-CaMKIIα-hM3Dq-EGFP (5.89E + 12 vg/mL, PT-0525), AAV2/9-CaMKIIα-hM4Di-EGFP (5.16E + 12 vg/mL, PT-0524) and CNO used in this study were purchased and verified by BrainVTA (Wuhan, China).

### Surgery and virus injection

Mice were anesthetized by intraperitoneal injections of pentobarbital (60 mg/kg, China National Pharmaceutical Group Corporation, Beijing, China) immediately before surgery, and further anesthesia was administered as needed based on the hind leg reflex. The eyes of mice were protected with Puralube Vet ointment (Chenxin Pharmaceuticals, Jining, China). Body temperature was maintained at a stable level by using a heating pad. Each mouse was fixed on the brain stereotaxic apparatus (RWD Life Science, Shenzhen, China), and the skull was leveled along the antero-posterior and medio-lateral axes (Zhao et al., 2022). A syringe pump (RWD Life Science, Shenzhen, China) was used to inject the virus. The total virus volume per injection site was 300 nL, at an injection speed of 30 nL/min. To avoid virus backflow, the pipette in place for 10 min after each injection and then carefully removed. Mice were placed on a heating pad after the surgery for recovery.

### 5-choice serial reaction time task (5-CSRTT)

The 5-CSRTT was managed through a touchscreen-based automated operating system. The apparatus consisted of a Bussey-Saksida mouse touchscreen chamber (Lafayette Instrument, IN, USA) with a chamber light, five stimulus response apertures light (4 cm × 4 cm) and a reward port containing a reward magazine with an infrared sensor for detection of a mouse entering into the port. Over 5 consecutive days, the mice were trained to perform tasks in the operation cage to obtain rewards. ABET II and Whisker Server software (Lafayette Instrument, IN, USA) were used to control the operating system and data collection.

### Pre-training

Before training, mice went through four adaptation stages. In the first stage, the mouse freely explored the operation box while the aperture light and the reward magazine light were off and no reward was given (10 min). In the second stage, the aperture light was off, the reward magazine light was on, and the mice were given liquid rewards (2% sucrose) after touching the reward magazine (30 min, 100 trials). In the third stage, one of the five stimulus response apertures was randomly switched on. When the mouse touched the screen, the reward magazine light was switched on, and the sucrose was immediately dispensed as a reward. Then, an aperture was randomly switched on (no interval time) to enter the next cycle (30 min, 100 trials). In the fourth stage, the basic procedure was the same as that in the third stage, except the next cycle started after a 5 s interval after the reward was given (30 min, 100 trials).

### Training

The mouse entered the training stage after completing the pre-training. The task difficulty gradually increases from Stages 1 to 6. To move from one stage to the next, each animal’s behavior had to stabilize at specific performance criteria (Table 1). The Stage 1 screen duration (SD), the duration of the light stimulus, was 30 s; the limited hold (LH), the length of time during which the animal could respond to the light stimulus, was 30 s; and the intertrial interval (ITI), the amount of time the animal had to wait before the light stimulus appeared, was 2 s. First, the reward magazine light was switched on, the mouse touched it, and the test was started 5-s timeout (TO). A stimulation aperture was randomly switched on; if the mouse touched the lighted stimulation aperture within the SD time, the sound feedback indicated that the touch was correct. At the same time, the reward magazine light was turned on; if the mouse touched the reward magazine during the LH time, it was rewarded with sucrose, and the next cycle started after the ITI.

**TABLE 1:** Schedule for stimulus parameters in the 5-CSRTT task.

Stage	Stage parameters			
	**SD(s)**	**LH(s)**	**ITI(s)**	**Progression criteria**
1	30	30	2	≥ 30 correct trials
2	20	20	2	≥ 30 correct trials
3	10	10	5	≥ 50 correct trials
4	5	5	5	≥ 50 correct trials; ≥75% accuracy; ≤ 25% omission
5	2.5	5	5	≥ 50 correct trials; ≥75% accuracy; ≤ 25% omission
6	1.8	5	5	≥ 50 correct trials; ≥75% accuracy; ≤ 25% omission

When the mouse responded prematurely, late, or incorrectly, a timeout penalty occurred, and the chamber light was turned on for 5 s. When the mouse reached the performance criteria for two consecutive days, the training program entered the next phase. The SD and LH were decreased, and the ITI was prolonged. See Table 1 for the subsequent process upgrade standards.

### Test

Once stable performance was achieved in Stage 6, the mouse entered the next test stage. In the *in vivo* fiber photometry experiment, Stage 4 was selected for testing to yield more incorrect, omission, and premature trials. In the chemogenetic experiment, a regular test (Stage 6: SD1.8-LH5-ITI5) and challenge test (LITI: SD1.8-LH5-ITI7) were selected for testing. Each test lasted 30 min.

### Fiber photometry recording

In the *in vivo* fiber photometry experiment, each mouse received a stereotactic injection of the virus into the right insular cortex [ + 2.22 anteroposterior (AP), + 2.3 mediolateral (ML), −1.75 dorsoventral (DV)]. Then the optical fiber was implanted [ + 2.22 anteroposterior (AP), + 2.3 mediolateral (ML), −1.72 dorsoventral (DV)], reinforced with three stainless-steel screws, and fixed with dental cement mixed with 502 glue. During the day of testing, the implanted fiber was connected via an external fiber to an integrated fiber optic recording device (RWD Life Science, Shenzhen, China). GCaMP6s fluorescence was bandpass filtered and collected by a photomultiplier tube using a 488 nm diode laser (OBIS 488LS, Coherent) coupled to an optical fiber. The current in the photomultiplier tube was amplified into voltage signals, which were then further filtered through a low-pass filter (30 Hz). Sampling was performed at 500 Hz using a data acquisition card (USB6009, National Instrument) using the software provided by RWD. The data for the individual trial of event stimulations were analyzed, and the values of fluorescence change (ΔF/F) were derived by calculating (F-F_0_)/F_0_. F_0_ was the baseline fluorescence level calculated by averaging the signals over the 2 s before the onset of stimulation.

### Chemogenetic manipulation

For chemogenetic inhibition or activation experiments, the mice were divided into two groups by injection with EGFP (saline/CNO) and hM4Di or hM3Dq (saline/CNO) virus. Mice were treated on the first day with saline and on the following day with CNO (*n* = 12 mice for each group). The virus was injected into the bilateral insula [ + 2.22 anteroposterior (AP), ± 2.3 mediolateral (ML), −1.75 dorsoventral (DV)] of each mouse. One week following the injection of the virus, the behavior experiment was conducted. After approximately 2 months of training, the mice were tested when their behavior was stable. To examine behavioral performance under varying degrees of task difficulty, we systematically varied the duration of the ITI. The ITI was set at 5 s in a regular test, whereas it was increased to 7 s in the challenge test. Intraperitoneal injections of CNO (0.3 mg/kg) or saline were conducted 30 min before the mice were subjected to regular testing in the 5-CSRTT chambers. One week after regular training and recovery, the mice underwent a challenge test in the 5-CSRTT chambers 30 min after CNO or saline injection.

### Histology and immunofluorescence staining

Mice were anesthetized with 1% (wt/vol) sodium pentobarbital (60 mg/kg) and perfused through the left cardiac ventricle with 0.9% NaCl, followed by 4% paraformaldehyde (PFA) (Solarbio, Cat# P1110). Brains were removed and postfixed overnight at 4°C, cryoprotected in 15 and 30% sucrose (Sigma-Aldrich, 57-50-1) for 24 h at 4°C, and then embedded into optimal cutting temperature (O.C.T.) (SAKURA, #4583). Serial coronal sections (35 μm thick) were prepared using a freezing microtome (Thermo Fisher Scientific Inc., Waltham, MA, USA). The brain slices were mounted on slides and dried at room temperature for 30 min (Chen et al., 2022; Yang et al., 2023).

Brain tissue slides were rehydrated with PBS for 15 min, washed with PBST (PBS + 1% Triton X-100) for 25 min, blocked with PBS containing 3% bovine serum albumin (Sigma-Aldrich, #1933) and 0.3% Triton X-100 for 1 h at room temperature and incubated with primary antibodies (diluted in 3% BSA) overnight at 4°C. Primary antibodies and dilutions (diluted in 3% BSA with 0.3% PBST) were as follows: anti-c-Fos (Abcam, ab190289, 1:1000) and anti-CaMKIIα (Santa Cruz Biotechnology, sc-13141, 1:50). The slides were washed in PBS three times for 5 min each time and incubated with fluorescent secondary antibodies (diluted in PBST and 3% BSA) for 1 h at room temperature. The following secondary antibodies were used at the indicated dilutions: Alexa Fluor 488-conjugated goat anti-rabbit IgG (Biotium, 20012, 1:500) and Alexa Fluor 568-conjugated goat anti-mouse IgG (Biotium, 20101, 1:500). Following six washes with PBS for 5 min each time, sections were counterstained with DAPI (ZSGB-BIO, ZLI-9557). For c-Fos staining, mice were perfused 45 min after behavioral experiments. Images were acquired with an Olympus FV-1200 confocal microscope (Olympus, Center Valley, PA, USA) and analyzed with Imaris 9.3.1 software (Abingdon, Oxfordshire, UK).

### Statistical analysis

All results are presented as the mean ± SEM and were analyzed by GraphPad Prism 8.0.2 (San Diego, CA, USA). ImageJ was used for statistical analysis of the positive cell numbers per unit area of immunofluorescence images. MATLAB (MathWorks, MA, USA) was used to process the *in vivo* fiber photometry experiment. Behavioral tests were analyzed by ANOVA (specifically stated in figures) followed by Tukey’s multiple comparisons test (Only the mice that had successfully reached the final stated progression criteria for Stage 6 and mice that exhibited normal locomotor activity following post-CNO treatment were eligible for statistical analysis). The c-Fos results were evaluated by two-tailed unpaired *t*-tests. The area under the curve (AUC) was analyzed by one-way ANOVA *p* < 0.05 was considered statistically significant.

## Results

### Establishment of the 5-CSRTT behavioral paradigm in mice

**FIGURE 1:**
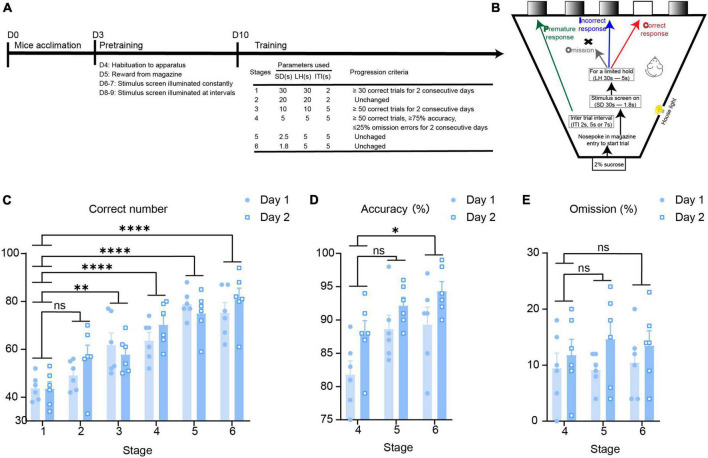
Establishment of the 5-CSRTT behavioral paradigm in mice. **(A)** The timeline of the 5-CSRTT. **(B)** The process of the 5-CSRTT. **(C–E)** 5-CSRTT behavioral data evaluation *n* = 6. Data are presented as the mean ± SEM. *p < 0.05, **p < 0.01, ****p < 0.0001, ns: no significance.

According to the mature 5-CSRTT behavioral paradigm, we constructed a mouse 5-CSRTT model (Humby et al., 1999). Male C57BL/6 mice aged 7–8 weeks were selected as experimental mice. Before the experiment began, 3 days of environmental acclimation were carried out, and then the mice underwent four stages of pre-training. After the mice completed the pre-training, they entered the training stage. This stage was progressive in difficulty; the SD time was shortened and the ITI time was prolonged with training, and the change in parameters requires the mice to pay more attention (Figure 1A). This paradigm evaluates the attention of mice by four behavioral indicators, namely, correct responses, incorrect responses, premature responses, and omission responses (Figure 1B). We performed statistical analysis on the behavioral indicators of mice after completing the 5-CSRTT, and the results showed that as the stage progressed, the correct response number (repeated-measures ANOVA: Stage 1 vs. Stage 2, adjusted *p* = 0.22; Stage 1 vs. Stage 3, adjusted *p* = 0.004; Stage 1 vs. Stage 4, adjusted *p* < 0.0001; Stage 1 vs. Stage 5, adjusted *p* < 0.0001; Stage 1 vs. Stage 6, adjusted *p* < 0.0001; Figure 1C) increased, and the accuracy rate (repeated-measures ANOVA: Stage 4 vs. Stage 5, adjusted *p* = 0.053; Stage 4 vs. Stage 6, adjusted *p* = 0.015; Figure 1D) increased accordingly when the mice performed the task, while there was no difference in the number of omission response (repeated-measures ANOVA: Stage 4 vs. Stage 5, adjusted *p* = 0.92; Stage 4 vs. Stage 6, adjusted *p* = 0.91; Figure 1E). The results indicate that we successfully constructed the mouse 5-CSRTT paradigm and that the mice could finish the 5-CSRTT program in a relatively stable manner.

### Identification of c-Fos + CaMKIIα neurons in the aIC related to attention

**FIGURE 2:**
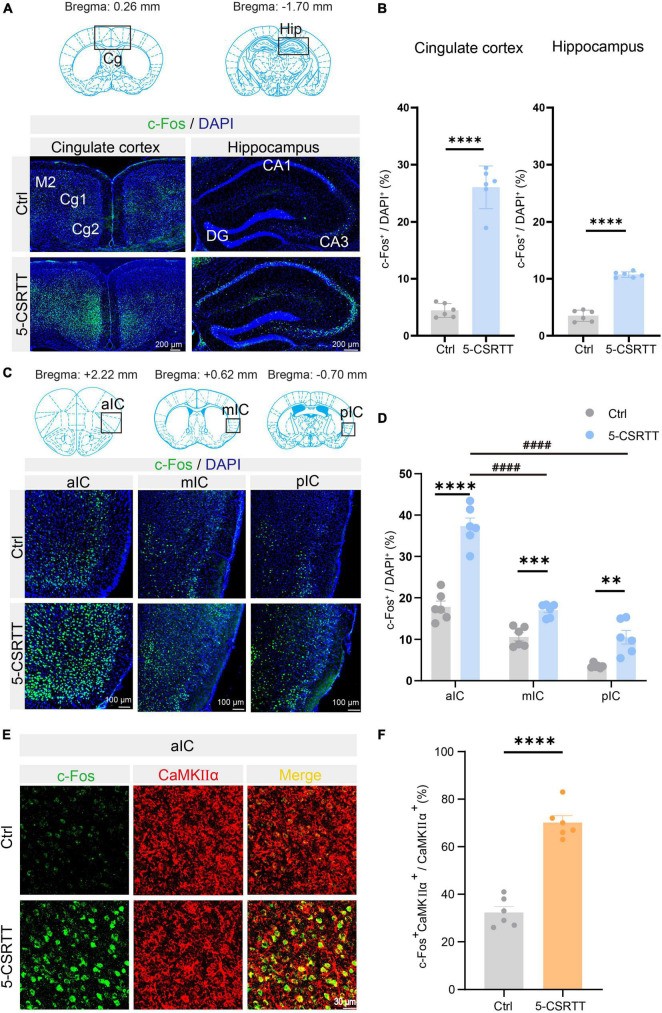
Identification of c-Fos + CaMKIIα neurons in the aIC related to attention. **(A)** Representative figures showing the expression of c-Fos in the cingulate cortex and hippocampus. Scale bar = 200 μm. **(B)** Quantitative statistics of the percentage of c-Fos-positive cells in the cingulate cortex and hippocampus *n* = 6. **(C)** Representative figures showing the expression of c-Fos in the aIC, mIC, and pIC. Scale bar = 100 μm. **(D)** Quantitative statistics of the percentage of c-Fos-positive cells in the aIC, mIC, and pIC *n* = 6. **(E)** Representative figure showing the colocalization of c-Fos + and CaMKIIα + cells in the aIC. Scale bar = 20 μm. **(F)** Percentage of c-Fos neurons colabeled with CaMKIIα, which is calculated as (CaMKIIα^+^c-Fos^+^/CaMKIIα^+^) *100% *n* = 6. Data are presented as the mean ± SEM. **p < 0.01, ***p < 0.001, ****p < 0.0001, ^####^*p* < 0.0001.

To identify the attention behavior-activated brain nuclei, we examined c-Fos expression in the brains of mice that had completed the 5-CSRTT by immunofluorescence staining. c-Fos is an immediate early gene commonly used as a marker for activated neurons. Previous studies have shown that the cingulate cortex (Passetti et al., 2002; Chudasama et al., 2003; Starski et al., 2019) and hippocampus (Li et al., 2018) are involved in the attention process in a 5-CSRTT mouse model. In this study, immunofluorescence staining showed that c-Fos in the 5-CSRTT group was significantly increased compared with control mice, which were also placed into the 5-CSRTT device but did not perform any attention behavioral test, in the cingulate cortex [unpaired two-tailed *t*-test: Ctrl vs. 5-CSRTT, t_(10)_ = 13.53, *p* < 0.0001] and hippocampus [unpaired two-tailed *t*-test: Ctrl vs. 5-CSRTT, t_(10)_ = 15.92, *p* < 0.0001; Figures 2A, B]. This suggests that increased numbers of c-Fos-activated cells may be a significant predictor of attention processes in the 5-CSRTT mouse model. Compared with control mice, the expression of c-Fos in the insular cortex was also significantly increased after completing the 5-CSRTT.

As previously described, the insular cortex is divided into three subregions, the aIC, mIC, and pIC, which are not completely consistent in physiological function. We first analyzed the c-Fos-positive rate in the aIC, mIC and pIC in mice that completed the 5-CSRTT. We found that compared with control mice, the c-Fos + neurons in the aIC region in 5-CSRTT mice were significantly increased [unpaired two-tailed *t*-test: Ctrl vs. 5-CSRTT, t_(10)_ = 8.23, *p* < 0.0001; Figures 2C, D]. The expression of c-Fos in the mIC was significantly higher than that in control mice [unpaired two-tailed *t*-test: Ctrl vs. 5-CSRTT, t_(10)_ = 5.47, *p* = 0.0003; Figures 2C, D]. Similarly, c-Fos expression in the pIC was also significantly increased after completing the 5-CSRTT [unpaired two-tailed *t*-test: Ctrl vs. 5-CSRTT, t_(10)_ = 4.20, *p* = 0.002; Figures 2C, D].

Although the staining results revealed that c-Fos expression in all three subregions of the insular cortex was increased, the statistical results showed that more neurons were activated in the aIC than in the mIC (one-way ANOVA: aIC vs. mIC, adjusted *p* < 0.0001; Figure 2D) and pIC (one-way ANOVA: aIC vs. pIC, adjusted *p* < 0.0001; Figure 2D). Previous studies have indicated that projection neurons in the cortex are primarily glutamatergic and express CaMKIIα (Liu and Murray, 2012), so we determined whether the neurons expressing c-Fos in the aIC were CaMKIIα neurons by colabeling c-Fos with CaMKIIα (Figure 2E). We found that the ratio of CaMKIIα and c-Fos double-positive neurons among CaMKIIα^+^ neurons (CaMKIIα^+^c-Fos^+^/CaMKIIα^+^) in control mice was significantly lower than that in the 5-CSRTT group [unpaired two-tailed *t*-test: Ctrl vs. 5-CSRTT, t_(10)_ = 9.95, *p* < 0.0001; Figure 2F]. This suggests that CaMKIIα neurons in the aIC were activated by the selective attention process of the 5-CSRTT.

### Activated aIC CaMKIIα neurons in response to attention

**FIGURE 3:**
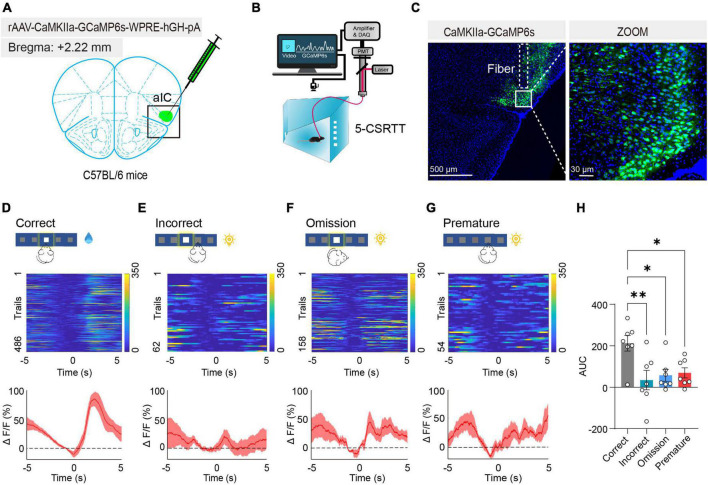
Activated aIC CaMKIIα neurons in response to attention. **(A)** Microinjection of CaMKIIα-GCaMP6s into the aIC. **(B)** The process of the 5-CSRTT using *in vivo* fiber photometry. **(C)** Representative figure showing GCaMP6s expression in the aIC. Scale bars, 500 and 30 μm. **(D–G)** Heatmap and ΔF/F of aIC Ca^2+^ signals in correct responses, incorrect responses, premature responses, and omission responses, respectively *n* = 7. **(H)** Quantification of the AUC in four behavioral indicators. Data are presented as the mean ± SEM. *p < 0.05, **p < 0.01.

To further confirm the relationship between the activated aIC CaMKIIα neurons and attention, we monitored neuronal activity using *in vivo* fiber photometry recording, which records the transient intracellular calcium (Ca^2+^) levels in behaving mice. We expressed the calcium indicator GCaMP6s in the aIC, which preferentially targets putative CaMKIIα neurons by CaMKIIα promoter-driven AAV transduction (Figure 3A). Next, an optical fiber was surgically implanted directly above the aIC nucleus, allowing us to record neuronal activity within the aIC by detecting GCaMP6s fluorescent signaling, and then we recorded the calcium photometry signals in mice during the 5-CSRTT (Figure 3B). The expression of GCaMP6s and the position of the optical fiber were further confirmed by histological analysis following each experiment (Figure 3C). Fiber photometry recordings were applied to four behavioral indicators: correct responses, incorrect responses, premature responses, and omission responses. Compared with the other three groups of behavioral indicators, the intracellular Ca^2+^ activity of aIC CaMKIIα neurons was significantly increased when the mice gave the correct response in the 5-CSRTT (Figures 3D–G). Summarizing the transient calcium levels of different behavioral indicators, the area under the curve (AUC) displayed a significant increase in correct responses when compared with the other three indicators (one-way ANOVA: Correct vs. Incorrect, adjusted *p* = 0.005; Correct vs. Omission, adjusted *p* = 0.02; Correct vs. Premature, adjusted *p* = 0.03; Figure 3H). Together, these results demonstrated that aIC CaMKIIα neurons were activated during the attentional process in mice.

### Chemogenetic inhibition of aIC CaMKIIα neurons disrupts attention

**FIGURE 4:**
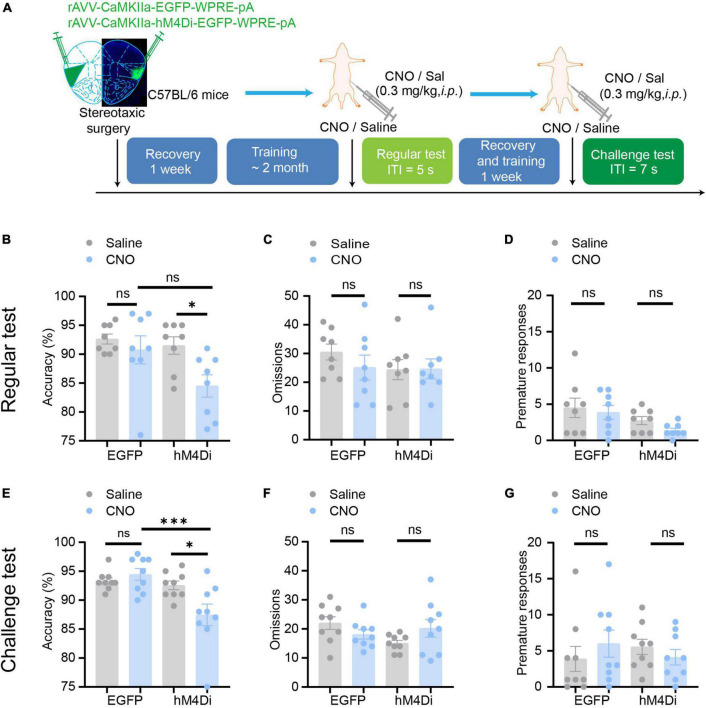
Chemogenetic inhibition of aIC CaMKIIα neurons disrupts attention. **(A)** The process of the 5-CSRTT. Three parameters, including accuracy, omissions, and premature responses, were analyzed. **(B–D)** In the regular test, chemogenetic inhibition of aIC CaMKIIα neurons decreased accuracy but did not affect premature responses or omissions *n* = 8 (EGFP) and 8 (hM4Di). **(E–G)** In the challenge test, chemogenetic inhibition of aIC CaMKIIα neurons decreased accuracy but did not affect premature responses or omissions *n* = 9 (EGFP) and 9 (hM4Di). Data are presented as the mean ± SEM. *p < 0.05, ***p < 0.001, ns: no significance.

To further examine the role of aIC CaMKIIα neurons in attention behavior, we used the DREADD chemogenetic technique to manipulate the activity of insular CaMKIIα neurons in mice. We used a stereotaxic injection instrument to inject the chemogenetic inhibitory virus rAAV- CaMKIIα-hM4Di-EGFP into the bilateral insula of mice, and rAAV- CaMKIIα-EGFP virus was used as a negative control. The mice then performed the 5-CSRTT behavioral training 1 week after virus injection. All experimental mice were restricted from drinking water according to the standard protocol, and they were free to drink water for 15 min after training or testing every day. We compared three parameters in the 5-CSRTT, including accuracy (correct responses/total responses) of attention, omissions of attention, and premature responses to measure impulsivity between control and chemogenetically suppressed mice. The overall activity of the aIC was modulated by CNO (0.3 mg/kg), which was intraperitoneally injected into the mice 30 min before the start of the test. After the behavioral test was completed, the mice were perfused, and brain tissue was cryosectioned to validate the location accuracy of virus injection and expression (Figure 4A).

In a regular test of the 5-CSRTT, during which the intertrial interval (ITI) was set at 5 s as a baseline, we found that chemogenetic inhibition of aIC CaMKIIα neurons dramatically decreased the accuracy of correct responses [two-way ANOVA; virus factor: *F*_(1_, _28)_ = 6.236, *p* = 0.02; drug factor: *F*_(1_, _28)_ = 4.306, *p* = 0.047; interaction: *F*_(1_, _28)_ = 2.079, *p* = 0.16; hM4Di-saline vs. hM4Di-CNO: adjusted *p* < 0.05; Figure 4B] but had no effect on the omission responses [two-way ANOVA; virus factor: *F*_(1_, _28)_ = 0.5167, *p* = 0.48; drug factor: *F*_(1_, _28)_ = 0.8634, *p* = 0.36; interaction: *F*_(1_, _28)_ = 0.6224, *p* = 0.44; Figure 4C] or premature responses [two-way ANOVA; virus factor: *F*_(1_, _28)_ = 1.271, *p* = 0.27; drug factor: *F*_(1_, _28)_ = 5.739, *p* = 0.02; interaction: *F*_(1_, _28)_ = 0.1787, *p* = 0.68; hM4Di-saline vs. hM4Di-CNO: adjusted *p* = 0.69; Figure 4D]. In addition, CNO treatment did not affect the behaviors of mice microinjected with the rAAV- CaMKIIα-EGFP control virus [two-way ANOVA; accuracy: virus factor: *F*_(1_, _28)_ = 6.236, *p* = 0.02; drug factor: *F*_(1_, _28)_ = 4.306, *p* = 0.047; interaction: *F*_(1_, _28)_ = 2.079, *p* = 0.16; EGFP-saline vs. EGFP-CNO: adjusted *p* = 0.88; omission: virus factor: *F*_(1_, _28)_ = 0.5167, *p* = 0.48; drug factor: *F*_(1_, _28)_ = 0.8634, *p* = 0.36; interaction: *F*_(1_, _28)_ = 0.6224, *p* = 0.44; premature: virus factor: *F*_(1_, _28)_ = 1.271, *p* = 0.27; drug factor: *F*_(1_, _28)_ = 5.739, *p* = 0.02; interaction: *F*_(1_, _28)_ = 0.1787, *p* = 0.68; EGFP-saline vs. EGFP-CNO: adjusted *p* = 0.96; Figures 4B–D]. To further confirm the role of aIC CaMKIIα neurons in the regulation of attention, we performed a challenge test with a longer ITI (ITI = 7 s). The results showed that accuracy [two-way ANOVA; virus factor: *F*_(1_, _32)_ = 2.964, *p* = 0.09; drug factor: *F*_(1_, _32)_ = 11.21, *p* = 0.002; interaction: *F*_(1_, _32)_ = 7.172, *p* = 0.012; hM4Di-saline vs. hM4Di-CNO: adjusted *p* < 0.05; EGFP-saline vs. EGFP-CNO: adjusted *p* = 0.91] was not affected in control mice treated with or without CNO but was significantly decreased in chemogenetically suppressed mice treated with CNO compared to saline (Figure 4E). Interestingly, the omission [two-way ANOVA; virus factor: *F*_(1_, _32)_ = 0.0846, *p* = 0.77; drug factor: *F*_(1_, _32)_ = 1.293, *p* = 0.26; interaction: *F*_(1_, _32)_ = 4.587, *p* = 0.04] and premature responses [two-way ANOVA; virus factor: *F*_(1_, _32)_ = 0.0505, *p* = 0.82; drug factor: *F*_(1_, _32)_ = 0.0056, *p* = 0.94; interaction: *F*_(1_, _32)_ = 1.436, *p* = 0.24] were not significantly different in either control or chemogenetically suppressed mice (Figures 4F, G). These results suggest that chemogenetic inhibition of aIC CaMKIIα neurons dramatically impairs attention in mice but does not affect impulsive behavior.

### Chemogenetic activation of aIC CaMKIIα neurons enhances attention

**FIGURE 5:**
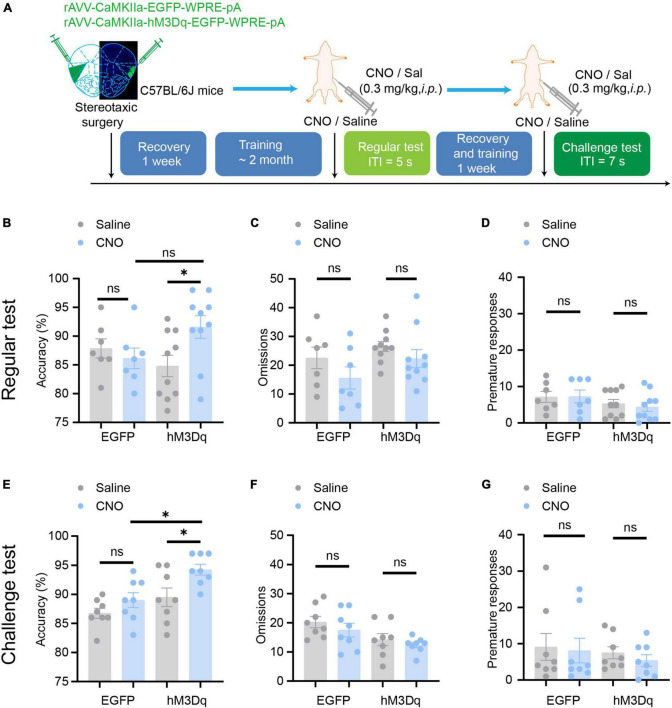
Chemogenetic activation of aIC CaMKIIα neurons enhances attention. **(A)** The process of the 5-CSRTT. **(B–D)** In the regular test, chemogenetic activation of aIC CaMKIIα neurons increased accuracy but did not affect premature responses or omissions *n* = 7 (EGFP) and 10 (hM3Dq). **(E–G)** In the challenge test, chemogenetic inhibition of aIC CaMKIIα neurons increased accuracy but did not affect premature responses or omissions *n* = 8 (EGFP) and 8 (hM3Dq). Data are presented as the mean ± SEM. *p < 0.05, ns: no significance.

We then tested whether aIC CaMKIIα neurons regulate attention bidirectionally. We used rAAV-CaMKIIα-hM3Dq-EGFP to selectively activate aIC neurons and then detected the effect on the 5-CSRTT following a similar paradigm as described above (Figure 5A). The mice were injected with the AAV on both sides of the aIC, and a week after surgical recovery, behavioral training was carried out. When the stable baseline was reached, we performed regular tests or challenge tests, and then studied the effects of chemogenetic activation of aIC neurons on attention behavior. In the regular test, we found that chemogenetic activation of aIC CaMKIIα neurons significantly increased the accuracy of correct responses [two-way ANOVA; virus factor: *F*_(1_, _30)_ = 1.768, *p* = 0.19; drug factor: *F*_(1_, _30)_ = 0.3937, *p* = 0.54; interaction: *F*_(1_, _30)_ = 4.955, *p* = 0.03; hM3Dq-saline vs. hM3Dq-CNO: adjusted *p* < 0.05; EGFP-saline vs. EGFP-CNO: adjusted *p* = 0.94] in the 5-CSRTT (Figure 5B), while there were no significant differences in omission [two-way ANOVA; virus factor: *F*_(1_, _30)_ = 3.266, *p* = 0.08; drug factor: *F*_(1_, _30)_ = 2.957, *p* = 0.096; interaction: *F*_(1_, _30)_ = 0.2041, *p* = 0.65] or premature responses [two-way ANOVA; virus factor: *F*_(1_, _30)_ = 0.0733, *p* = 0.79; drug factor: *F*_(1_, _30)_ = 2.861, *p* = 0.101; interaction: *F*_(1_, _30)_ = 0.1391, *p* = 0.71] (Figures 5C, D). In the challenge session (ITI = 7 s), the mice with chemogenetic activation of aIC CaMKIIα neurons showed significantly higher accuracy of correct responses [two-way ANOVA; virus factor: *F*_(1_,_28)_ = 8.495, *p* = 0.007; drug factor: *F*_(1_, _28)_ = 11.10, *p* = 0.002; interaction: *F*_(1_,_28)_ = 1.084, *p* = 0.307; hM3Dq-saline vs. hM3Dq-CNO: adjusted *p* < 0.05; EGFP-saline vs. EGFP-CNO: adjusted *p* = 0.56; hM3Dq-CNO vs. EGFP-CNO: adjusted *p* < 0.05] than control mice (Figure 5E), but the omission [two-way ANOVA; virus factor: *F*_(1_,_28)_ = 1.496, *p* = 0.23; drug factor: *F*_(1_, _28)_ = 8.654, *p* = 0.007; interaction: *F*_(1_,_28)_ = 0.535, *p* = 0.82; hM3Dq-saline vs. hM3Dq-CNO: adjusted *p* = 0.896; EGFP-saline vs. EGFP-CNO: adjusted *p* = 0.73] and premature responses [two-way ANOVA; virus factor: *F*_(1_,_28)_ = 0.3199, *p* = 0.58; drug factor: *F*_(1_, _28)_ = 0.6270, *p* = 0.44; interaction: *F*_(1_,_28)_ = 0.0415, *p* = 0.84] were not affected (Figures 5F, G). These results suggest that chemogenetic activation of aIC CaMKIIα neurons is important to promote attention performance without affecting impulsive behavior. Our findings, in conjunction with chemogenetic the inhibition results, offer conclusive proof that aIC CaMKIIα neurons play a crucial role in regulating attentional accuracy in mice.

## Discussion

Previous findings have confirmed that the aIC subregion, associated with limbic areas comprising the salience network, is critical for cognitive functions, such as behavioral motivation, that are compromised in drug addiction (Deng et al., 2021). However, its role in attention is still elusive. In this study, with the establishment the of 5-CSRTT behavioral paradigm in mice, we identified the specific activation of aIC neurons in the 5-CSRTT through both c-Fos immunofluorescence staining and fiber photometry recording. Chemogenetic inhibition of aIC CaMKIIα neurons disrupted attention behavior; in contrast, chemogenetic activation of aIC CaMKIIα neurons elevated the accuracy of correct responses in mice. Together, our results indicated the important role of aIC CaMKIIα neurons in regulating attention.

Attention is a complex core cognitive function responsible for prioritizing the selection of internal and/or external sensory stimuli for further processing (Katsuki and Constantinidis, 2014). Attention disorder is a characteristic of many mental diseases, such as attention deficit hyperactivity disorder (ADHD), autism spectrum disorder (ASD) and bipolar disorder (BP). The 5-CSRTT is widely recognized as a valuable test of attention in animals in cognitive and behavioral research. This test can detect multiple aspects of cognition at the same time, including attention and impulsive behavior, making it particularly valuable to study cognitive function and cognitive impairment in various neurological and psychiatric diseases (Robbins, 2002; Boutros et al., 2017). Therefore, the well-established 5-CSRTT paradigm in mice provides us with a reliable model to screen attention-related brain areas, which are also of great value in the diagnosis and treatment of attention deficit-related disorders.

Accordingly, attention is a process that requires the participation of multiple brain regions. Accumulating data indicate that many brain regions are involved in the attention process in the 5-CSRTT mouse model. For instance, it has been demonstrated that accuracy is decreased as a consequence of excitotoxic lesions to the medial prefrontal cortex (Muir, 1996). Complementary findings demonstrated that lesions in particular subregions of the prefrontal cortex differentially impact task-related behavioral measures. Several studies that focused specifically on particular brain regions have identified some anatomical regions that are involved in particular aspects of attention and inhibitory regulation. For example, accuracy decreases as a result of lesions in the dorsal pregenual (or supragenual) anterior cingulate cortex (ACC) subregion (Passetti et al., 2002; Chudasama et al., 2003). Lesions in the ventral infralimbic cortex also cause more premature reactions (Chudasama et al., 2003). In addition, a recent study using multiple genetic and optogenetic approaches has provided evidence that serotonin receptor 2c-expressing cells in the ventral CA1 control attention via innervation of the Edinger-Westphal nucleus (Li et al., 2018). c-Fos is often associated with the excitatory activity of neurons and is defined as a marker of neuronal activation (Hiroi et al., 1997; Kim et al., 2012). In this study, compared with control mice, the insular neurons of mice that had just completed the 5-CSRTT were significantly activated with a significantly upregulated number of c-Fos + cells, suggesting that the insula responds to attention behavior to some extent in mice. However, the 5-CSRTT is a multifaceted paradigm that involves various processes including motoric, sensory, and reward, etc. (Bari et al., 2008). Our current study cannot exclude the aforementioned confounding factors, and future investigations should be conducted in a more detailed and systematic manner.

The insula is an important part of the limbic cortical system (Mesulam and Mufson, 1982). Previous studies have shown that the insula is not only involved in emotional processing (Liu et al., 2018) and perception acquisition (Gal-Ben-Ari and Rosenblum, 2011) but also related to the cognitive processing of high-level emotions (Craig, 2009; Nieuwenhuys, 2012). The IC is divided into three subregions of the same size, namely, the anterior insular cortex (aIC), medial insular cortex (mIC), and posterior insular cortex (pIC). The aIC is mainly related to cognition, while the mIC and pIC are mainly related to the recognition process. The aIC, related to interoceptive representations (Paulus and Stewart, 2014), deals with all subjective feelings of physical and emotional consciousness and is implicated in executive functions and impulse control processes, such as decision-making under risk (Naqvi et al., 2014) and specific motivational functions (Deng et al., 2021). These results indicate that the aIC might also play a certain role in regulating attention. In our current study, we only explored the influence of the aIC on attention, and the mechanism of whether and how the mIC and pIC participate in the regulation of attention still needs to be further elucidated. Furthermore, our immunofluorescent results demonstrate that colocalization of c-Fos with CaMKIIα staining in 5-CSRTT mice indicated that the majority of the aIC CaMKIIα neurons were activated. Previous studies have demonstrated that most cortex neurons are projection neurons, the insula also contains several interneurons (Ramos-Prats et al., 2022). Altogether, it is still necessary to explore the potential role of those interneurons in the insular cortex in regulating attention in the future.

The majority of earlier lesion studies in humans showed interoceptive deficits with in patients with aIC lesions (Ronchi et al., 2015; Terasawa et al., 2015; García-Cordero et al., 2016; Critchley and Garfinkel, 2017), and the results from patients who had focal insular damage added to the evidence that the aIC has an important function in interoceptive attention. Patients with aIC lesions had lower interoceptive attention accuracy and sensitivity than non-insular lesion patients and healthy controls. In this study, compared with control mice, inhibition or activation of aIC CaMKIIα neurons significantly reduced or enhanced attention accuracy, suggesting that aIC lesions play a role not only in interoceptive attention but also in visuospatial attention. Furthermore, given that a previous study has demonstrated that long-ITI sessions allow for a more comprehensive assessment of attention (Oliver et al., 2009), we performed regular tests and challenge tests. Surprisingly, our results showed that similar numbers of accuracy, omission, and premature responses were observed in both the regular test and challenge test, which may indicate that the degree of task difficulty had no effect on mouse behavior in our study. It should also be noted that only male mice were used in our study, and the consistency of the results in female mice needs to be further explored.

In summary, our study demonstrated that aIC CaMKIIα neurons play an important role in the regulation of attention, and expanded the understanding of the role of the insula in cognitive function. Our findings may also shed light on the mechanism underlying attention deficiency-related disorders. Notably, attention relies on multiple brain regions and circuits. Although we only analyzed the role of CaMKIIα neurons within the aIC as a critical functional hub in regulating attention, the detailed role and mechanisms of aIC-mediated neural circuits, including the afferent and efferent connections throughout the entire brain, still need further investigation.

## Data availability statement

The original contributions presented in the study are included in the article/supplementary material, further inquiries can be directed to the corresponding author.

## Ethics statement

The animal study was reviewed and approved by the Institutional Animal Care and Use Committee of the Beijing Institute of Basic Medical Sciences.

## Author contributions

HW: conceptualization, writing—review and editing, visualization, supervision, project administration, and funding acquisition. YM, SJ, XZ, SL, LC, ZZ, WS, and YW: methodology. YM, SJ, and XZ: software. YM, SJ, and HW: formal analysis. YM: writing—original draft preparation. All authors have read and agreed to the published version of the manuscript.
